# Meeting physical activity guidelines in conjunction with higher protein intake: associations with appendicular lean soft tissue index in middle aged adults with cancer

**DOI:** 10.1007/s00520-025-10290-6

**Published:** 2026-01-06

**Authors:** Konstantinos Prokopidis, Stefano Cacciatore, Nicola Veronese, Brendon Stubbs, Paolo Piaggi, John A. Batsis, Carla M. Prado, Mathias Schlögl

**Affiliations:** 1 Department of Musculoskeletal and Ageing Science, Institute of Life Course and Medical Sciences, University of Liverpool, Liverpool, L7 8TX UK; 2 Department of Geriatrics, Orthopedics and Rheumatology, Università Cattolica del Sacro Cuore, L.Go F. Vito 1, 00168 Rome, Italy; 3 Geriatric Unit, Department of Medicine, University of Palermo, Palermo, Italy; 4 Saint Camillus International University of Health Sciences, Rome, Italy; 5 Department of Psychological Medicine, Institute of Psychiatry, Psychology and Neuroscience (IoPPN), King’s College London, London, UK; 6 Department of Information Engineering, University of Pisa, Pisa, Italy; 7 Department of Nutrition, Gillings School of Global Public Health, University of North Carolina at Chapel Hill, Chapel Hill, NC USA; 8 Division of Geriatric Medicine, UNC School of Medicine, Chapel Hill, NC USA; 9 Department of Agricultural, Food & Nutritional Science, University of Alberta, Edmonton, Canada; 10 Clinic Barmelweid, Department for Geriatric Medicine, Erlinsbach, Switzerland; 11 Fondazione Policlinico Universitario “Agostino Gemelli” IRCCS, L.Go A. Gemelli 8, 00168 Rome, Italy

**Keywords:** Cancer, Sarcopenia, Muscle wasting, Protein intake, Nutrition

## Abstract

**Background:**

Loss of muscle mass is a common concern among patients with cancer. The aim of this study was to examine whether meeting the World Health Organization physical activity guidelines in combination with a higher vs. lower than the recommended daily allowance (RDA) protein intake is associated with greater appendicular lean soft tissue index (ALSTI) in adults aged 40–59 years with cancer from the National Health and Nutrition Examination Survey.

**Methods:**

Participants were categorized by physical activity levels (moderate ≥ 150 min/week or vigorous ≥ 75 min/week) and protein intake (> 0.8 vs. ≤ 0.8 g/kg/day) assessed via two interviewer-administered 24-h dietary recalls. ALSTI was calculated using dual-energy X-ray absorptiometry (kg/m^2^). Linear regression models estimated associations, adjusting for demographic, clinical, and dietary covariates.

**Results:**

Among 169 participants (mean age 51.0 ± 5.6 years; 69% women, mean ALSTI 7.74 ± 1.66 kg/m^2^), those meeting vigorous or moderate physical activity guidelines with higher protein intake did not show a significant association with ALSTI in the fully adjusted models (vigorous: β = 0.08, standard error (SE) 0.12, *p* = 0.53; moderate: β = -0.05, SE 0.15, *p* = 0.76). However, a significantly positive link was found in those meeting both vigorous and moderate physical activity (β = 0.40, b SE 0.02, p < 0.01).

**Conclusions:**

Meeting vigorous or moderate physical activity guidelines in combination with higher vs. lower protein intake was not associated with ALSTI in adults with cancer. However, meeting both was positively linked to ALSTI. Longitudinal and interventional studies using objective measures and longitudinal designs are needed to clarify the role of physical activity with adequate protein intake in preserving muscle health in this clinical population.

**Supplementary Information:**

The online version contains supplementary material available at 10.1007/s00520-025-10290-6.

## Introduction

During ageing, the maintenance of muscle mass and strength is essential for preserving functional independence and mitigating the risk and consequences of sarcopenia, a condition characterized by the progressive loss of skeletal muscle mass and strength [20]. Sarcopenia may present as primary, reflecting the physiological muscle loss associated with ageing, or as secondary, arising from or exacerbated by non-age-related factors such as chronic diseases, surgical interventions, malnutrition, or prolonged physical inactivity [9]. In this context, cancer, a condition whose prevalence is increased in older adults, poses additional challenges to muscle health. The effects of cancer treatments, such as chemotherapy, together with cancer-associated cachexia and anorexia, can markedly accelerate muscle wasting, compromise mobility, and substantially reduce quality of life [10, 16]. Among the modifiable determinants of impaired muscle health, optimal physical activity and nutritional intake emerge as key factors, particularly relevant in patients with cancer who are inherently more susceptible to rapid deterioration in muscle mass and function [34, 36].

The World Health Organization (WHO) recommends that adults should engage in at least 150–300 min per week of moderate intensity or at least 75 min per week of vigorous intensity physical activity, alongside muscle-strengthening exercises performed at least two times weekly [30]. Adherence to these guidelines may play a critical role in mitigating the muscle wasting effects often observed in individuals with cancer [2]. Appendicular lean soft tissue index (ALSTI), a measure of ALST normalized to height squared (kg/m^2^), is considered an accepted body composition marker, a proxy of muscle health, and is associated with better physical function, reduced risk of falls, and enhanced quality of life [15, 42]. Furthermore, adequate protein intake represents another cornerstone in the maintenance of muscle health, especially when combined with physical activity, due to its capacity of stimulating muscle protein synthesis (MPS). Ageing and catabolic conditions such as cancer are known to reduce the efficiency of MPS, thereby increasing dietary protein requirements of protein to preserve muscle tissue [29]. In particular, protein intakes ≥ 1.4 g/kg body weight have been proposed as a therapeutic target in cancer, aiming to attenuate muscle mass losses [3, 7].

Although the individual roles of physical activity and protein intake in preserving muscle health are well established, their combined influence, particularly in individuals with cancer, remains underexplored. In this context, evaluating whether meeting the WHO physical activity recommendations, in conjunction with a higher protein intake, is associated with improved ALSTI may offer valuable insights to inform pragmatic, lifestyle-based interventions aimed at preserving muscle mass and function in oncology care. In this study, we investigated the association between adherence to WHO-recommended levels of moderate or vigorous physical activity and a higher protein intake with ALSTI in middle-aged adults with cancer living in the United States.

## Methods

### Study design and data source

Data from consecutive cycles of the National Health and Nutrition Examination Survey (NHANES) database, specifically from 2007–2008 to 2017–2020, were pooled to analyse vigorous and moderate recreational activities. ALSTI data were collected from the 2001–2002 and 2011–2018 cycles, with dietary intake assessed via one 24-h dietary recall in NHANES cycles from 1999–2002 and two 24-h dietary recalls in NHANES cycles from 2003–2018. For participants with only one reliable dietary recall (NHANES 1999–2002), data were included as registered; for those with two reliable recalls (in later cycles), the average intake was used. The NHANES is a series of cross-sectional surveys conducted by the National Center for Health Statistics (NCHS) of the U.S. Centers for Disease Control and Prevention (CDC), which gathers data through interviews, physical exams, and laboratory tests. The NHANES database is publicly accessible via the NCHS website. The research protocol was approved by the NCHS Ethics Review Committee, and informed consent was obtained from all participants. The study was conducted in accordance with the ethical guidelines established by the Declaration of Helsinki and adhered to the standards set out in the STROBE Statement for reporting observational research [43].

### Study population and sample derivation

**Fig. 1:**
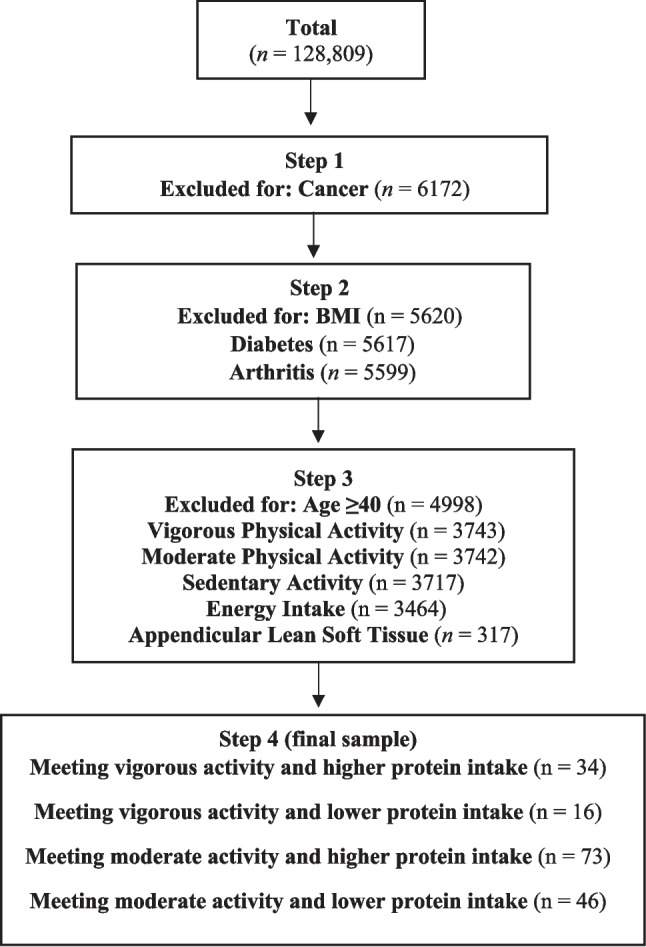
Flowchart of the included participants.

Participants aged 40–59 years who reported a prior cancer diagnosis and had complete data on physical activity, dietary intake, and ALSTI were included. This age range was selected to examine a younger and more metabolically homogeneous cancer-affected population, minimizing confounding from advanced age-related sarcopenia, multimorbidity, and functional decline. Cancer history was self-reported via the question: “Have you ever been told by a doctor or other health professional that you had cancer or a malignancy of any kind?” Among 128,809 individuals screened, 6172 reported a history of cancer, and 5599 had additional data on body mass index (BMI) and related comorbidities (i.e., arthritis, diabetes). After applying inclusion criteria, 317 patients with complete data were identified. Out of these, 34 (10.7%) and 16 (5.0%) individuals met the vigorous physical activity guidelines while consuming a higher and lower protein intake, respectively, while 73 (23.0%) and 46 (14.5%) met the moderate physical activity guidelines while consuming a higher and lower protein intake, respectively, providing in total 169 participants. A detailed flow diagram of participant selection is shown in Fig. 1.

### Baseline characteristics

Information was collected on race (Mexican, non-Hispanic White, non-Hispanic Black, Hispanic, and other groups) and education level (college or higher, college or associate degree, high school, or other), comorbidities (arthritis, diabetes). BMI was calculated by dividing weight by the square of height. During physical examinations at the Mobile Examination Centre (MEC), blood samples were collected and analysed for serum haemoglobin, albumin, and creatinine. Blood was drawn into tubes with ethylene diamine tetraacetic acid (EDTA) to prevent clotting, centrifuged to isolate serum, and stored at −70 °C before laboratory analysis. Serum creatinine levels were measured using the DxC800 modular chemistry analyzer, which applies the IDMS-standardized Jaffe rate method. Serum albumin concentration was assessed with the DxC800 using the bichromatic digital endpoint method with Bromcresol Purple (BCP) dye. Total serum cholesterol and high-density lipoprotein cholesterol (HDL-C) levels were determined using enzymatic methods.

### Exposure variables

#### Physical activity

Physical activity was assessed using the NHANES Physical Activity Questionnaire (PAQ), which captures self-reported participation in recreational moderate and vigorous activities through the question “How much time do you spend doing vigorous/moderate-intensity sports, fitness or recreational activities on a typical day?”. Participants were classified as meeting physical activity guidelines if they reported ≥ 150 min/week of moderate-intensity activity or ≥ 75 min/week of vigorous-intensity activity, following WHO recommendations [5]. Weekly duration was calculated by multiplying reported daily minutes by seven.

#### Protein intake and appendicular lean soft tissue

Protein intake was derived using two interviewer-administered 24-h dietary recalls. Average protein intake (g/day) was normalized to bodyweight (g/kg/day). Participants were stratified into "higher" and "lower" protein intake based on the recommended dietary allowance (RDA) of 0.8 g/kg/bodyweight. Nutrient composition was performed using USDA’s Food and Nutrient Database for Dietary Studies. Body composition was measured using dual-energy X-ray absorptiometry (DXA), with data analyzed through Hologic QDR-4500 software, version Apex 3.2 (Hologic, Bedford, MA, USA). ALSTI was calculated by summing the ALST (excluding bone mineral content) from both arms and legs. ALSTI assessments were limited to individuals aged up to 59 years. ALSTI was expressed in kg/m^2^.

### Statistical analysis

The Shapiro–Wilk test was applied to assess normality. Depending on the distribution, comparisons were performed using independent t-tests or Mann–Whitney U test. Categorical variables were compared using chi-squared tests. Linear regression was used to estimate the association between meeting moderate or vigorous physical activity guidelines, protein intake, and ALSTI. Both meeting moderate and vigorous activities, as well as higher or lower protein intake, were treated as categorical variables. ALSTI was treated as a continuous variable. To account for unequal selection probabilities, nonresponse bias, and oversampling of certain groups (e.g., racial minorities, elderly individuals, or low-income populations), sampling weights were applied. Additionally, appropriate stratum and cluster variables were employed to adjust variance estimates, ensuring accurate representation of the survey’s intricate design. Three models were estimated: an unadjusted model; Model 1, adjusted for age, sex, BMI, race, and education; and Model 2, additionally adjusted for arthritis, diabetes, and energy adjusted protein and alcohol intake as suggested previously [45]. This method adjusts for differences in total energy intake by computing residuals from a regression model where protein and alcohol intake (g/day), separately, are regressed on total energy intake (kcal/day). These residuals, reflecting energy adjusted protein and alcohol intake, were used in subsequent analyses to assess relationships with ALSTI in our fully adjusted model. Moreover, findings were reported as β coefficients with their standard errors (SE). To address multiple testing, we implemented the Bonferroni correction, modifying the significance level by dividing the alpha value by the number of tests performed. Statistical significance was set at p < 0.05. All analyses were conducted using IBM SPSS Statistics, version 29.0.

## Results

A total of 169 adults aged 40–59 years with a history of cancer met the inclusion criteria and reported engaging in either moderate or vigorous physical activity in accordance with WHO guidelines. Baseline characteristics, stratified by physical activity intensity and protein intake category, are presented in Table 1.

**Table 1:** Baseline characteristics based on physical activity and protein intake levels. Data is expressed as mean (standard deviation).

Outcomes	Meeting Vigorous Activity & Higher Protein *n* = 34	Meeting Vigorous Activity & Lower Protein *n* = 16	P value	Meeting Moderate Activity & Higher Protein *n *= 73	Meeting Moderate Activity & Lower Protein *n *= 46	P value
Sex (female)	20 (58.8)	**13 (81.3)**	0.20	**48 (65.8)**	**37 (80.4)**	0.10
Age (years)	51.7 (5.6)	50.8 (5.3)	0.61	51.2 (5.6)	50.3 (5.6)	0.35
Race n – (%)						
Hispanic	6 (17.6)	10 (62.5)	< 0.01*	13 (17.8)	17 (36.9)	0.23
Non-Hispanic White	20 (58.8)	3 (18.8)		47 (64.4)	22 (47.8)	
Non-Hispanic Black	5 (14.7)	2 (12.5)		7 (9.6)	4 (8.7)	
Other Race	3 (8.8)	1 (6.3)		6 (7.2)	3 (6.5)	
Education n – (%)						
College or above	21 (61.2)	6 (37.5)	0.32	37 (50.7)	12 (26.1)	0.12
College or associate degree	9 (26.5)	7 (43.8)		21 (28.8)	16 (34.8)	
High school	3 (12.1)	1 (6.3)		11 (15.1)	11 (23.9)	
Other (below high school)	1 (2.9)	2 (12.5)		4 (5.5)	7 (15.2)	
Bodyweight (kg)	73.1 (13.8)	96.2 (23.2)	< 0.01*	77.8 (21.3)	87.2 (18.7)	< 0.01*
Body mass index (kg/m^2^)	26.1 (4.5)	34.6 (8.1)	< 0.01*	28.2 (7.1)	32.4 (6.8)	< 0.01*
Arthritis n – (%)	5 (14.7)	9 (56.3)	< 0.01*	20 (27.4)	19 (41.3)	0.16
Diabetes n – (%)	1 (2.9)	2 (12.5)	0.15	7 (9.6)	9 (19.6)	0.04*
Vigorous activity (min/week)	359.7 (158.6)	400.3 (220.2)	0.46	111.9 (200.8)	93.6 (221.6)	0.64
Moderate activity (min/week)	292.4 (403.8)	205.6 (266.2)	0.44	458.8 (372.6)	533.4 (443.6)	0.33
Sedentary activity (min/day)	394.4 (243.6)	438.8 (195.6)	0.53	384.2 (210.2)	369.1 (180.2)	0.69
Energy intake (kcal/day)	2147 (743)	1412 (520)	< 0.01*	2213 (748)	1436 (553)	< 0.01*
Protein intake (g/day)	90.3 (36.6)	54.1 (16.7)	< 0.01*	92.9 (32.1)	50.5 (17.9)	< 0.01*
Protein intake (g/kg/bodyweight)	1.24 (0.42)	0.57 (0.15)	< 0.01*	1.23 (0.41)	0.58 (0.16)	< 0.01*
Carbohydrate intake (g/day)	242.7 (102.8)	171.9 (67.9)	0.02*	244.5 (104.8)	179.9 (79.6)	< 0.01*
Fibre intake (g/day)	18.8 (8.1)	11.4 (5.6)	< 0.01*	18.8 (7.4)	11.3 (6.9)	< 0.01*
Fat intake (g/day)	85.7 (36.8)	55.2 (28.6)	< 0.01*	91.1 (37.4)	53.0 (22.3)	< 0.01*
Alcohol intake (g/day)	10.3 (13.6)	4.0 (6.3)	0.08	10.1 (14.4)	8.1 (22.1)	0.54
Total cholesterol (mg/dL)	206.9 (83.2)	169.1 (52.6)	0.10	191.6 (49.5)	180.5 (72.2)	0.32
High-density lipoprotein cholesterol (mg/dL)	64.1 (28.1)	52.8 (21.2)	0.16	60.0 (24.3)	48.0 (19.9)	< 0.01*
Hemoglobin (g/L)	13.4 (2.8)	11.7 (3.7)	0.09	13.6 (2.2)	12.7 (3.2)	0.05
Serum albumin (g/L)	4.16 (0.78)	3.84 (1.05)	0.24	4.14 (0.76)	3.78 (1.22)	0.05
Appendicular lean soft tissue index (kg/m^2^)	7.41 (1.58)	8.65 (1.71)	0.02*	7.37 (1.56)	7.88 (1.75)	0.10

^*^Indicates significance at < 0.05

Among individuals meeting the vigorous activity guidelines, those with lower protein intake (0.57 ± 0.15 g/kg/bw) exhibited significantly higher body weight (96.2 ± 23.2 kg vs. 73.1 ± 13.8 kg, p < 0.01) and BMI (34.6 ± 8.1 kg/m^2^ vs. 26.1 ± 4.5 kg/m^2^, p < 0.01) compared to their higher protein intake counterparts (1.24 ± 0.42 g/kg/bw). They also showed a higher prevalence of arthritis (56.3% vs. 14.7%, p < 0.01) and a higher ALSTI (8.65 ± 1.71 vs. 7.41 ± 1.58 kg/m^2^, *p* = 0.02), despite significantly lower reported energy (1412 ± 520 vs. 2147 ± 743 kcal/day, p < 0.01) and protein intake (0.57 ± 0.15 vs. 1.24 ± 0.42 g/kg/day, p < 0.01). A similar trend was observed for fibre, fat, and carbohydrate intake, all lower in the low-protein subgroup. There were no significant differences in age, weekly vigorous or moderate activity duration, or serum albumin and hemoglobin levels between the two groups.

In the subgroup meeting moderate activity guidelines, individuals consuming lower protein (0.58 ± 0.16 g/kg/bw) also displayed significantly higher body weight and BMI (p < 0.01 for both), along with lower HDL-C (p < 0.01), lower energy and nutrient intake across all macronutrient categories, and a non-significant trend toward higher arthritis and diabetes prevalence vs. those in the higher protein intake group (1.23 ± 0.41 g/kg/bw). No statistically significant differences in ALSTI were observed between higher and lower protein intake groups in this stratum (7.88 ± 1.75 vs. 7.37 ± 1.56 kg/m^2^, *p* = 0.10).

### Association of meeting physical activity guidelines and consuming a protein intake above the RDA with ALSTI

When both vigorous and moderate physical activity were combined, a positive association between higher protein intake and ALSTI was found in the fully adjusted model (β = 0.40, SE 0.02, p < 0.01). Meeting vigorous physical activity guidelines combined with higher protein intake, in the unadjusted model, was not associated with ALSTI (*p* = 0.64), for which similar results were found about Model 2 (β = 0.08, SE 0.12, *p* = 0.53). Likewise, regarding moderate physical activity combined with higher than the RDA protein intake, no significant associations were found with ALSTI across models (unadjusted: β = 0.48, SE 0.45, *p* = 0.29; Model 1: β = −0.19, SE 0.13, *p* = 0.13; Model 2: β = −0.05, SE 0.15, *p* = 0.76). Linear regression analyses are presented in Table 2.

**Table 2:** Association of meeting vigorous and moderate physical activity guidelines and a higher vs. lower than RDA protein intake with appendicular lean soft tissue index.

**Moderate and vigorous physical activity**									
	**Unadjusted**			**Model 1**			**Model 2**		
**Outcome**	**p**	**b**	**SE**	**p**	**b**	**SE**	**p**	**b**	**SE**
Appendicular lean soft tissue index (kg/m^2^)	< 0.01*	−0.66	0.02	0.06	−0.44	0.11	< 0.01*	0.40	0.02
**Vigorous physical activity**									
	**Unadjusted**			**Model 1**			**Model 2**		
**Outcome**	**p**	**b**	**SE**	**p**	**b**	**SE**	**p**	**b**	**SE**
Appendicular lean soft tissue index (kg/m^2^)	0.64	0.23	0.48	< 0.01*	−0.40	0.14	0.53	0.08	0.12
**Moderate physical activity**									
	**Unadjusted**			**Model 1**			**Model 2**		
**Outcome**	**p**	**b**	**SE**	**p**	**b**	**SE**	**p**	**b**	**SE**
Appendicular lean soft tissue index (kg/m^2^)	0.29	0.48	0.45	0.13	−0.19	0.13	0.76	−0.05	0.15

^*^P < 0.05 indicates significance

Model 1: adjusted for age, sex, body mass index, race, and education

Model 2: adjusted for Model 1 and arthritis, diabetes, and energy adjusted protein and alcohol intake

## Discussion

The present analysis did not identify significant associations between adherence to WHO physical activity guidelines, separately, higher protein intake, and ALSTI in adults aged 40–59 years with history of cancer. However, meeting both vigorous and moderate physical activity was linked to a higher ALSTI. These findings may be divergent from prior research supporting the combination of physical activity and higher (than RDA) protein intakes for muscle mass preservation. In this context, vigorous activity alone may not provide sufficient anabolic stimulus, particularly in the absence of structured resistance training, which is considered more effective for inducing lean soft tissue gains. Systematic reviews and meta-analyses consistently demonstrate that resistance exercise, rather than aerobic or general vigorous activity, is required to induce significant improvements in skeletal muscle mass and strength in patients with cancer and survivors. For example, Kang et al*.* [19] found that resistance exercise interventions led to improvements in muscle mass and performance in adult patients with cancer, with 67–100% of studies reporting positive effects on muscle mass outcomes. Similarly, meta-analyses have shown that resistance training increases lean soft tissue by approximately 0.5–0.8 kg, whereas aerobic training alone provides smaller or inconsistent effects [8, 14]. These observations are consistent with position statements and guidelines from leading professional organizations. For example, the American College of Sports Medicine recommends resistance training as a core component of exercise prescriptions for cancer survivors to counteract muscle loss and improve functional outcomes [6]. Consistently, the American Society of Clinical Oncology also highlights the role of resistance exercise in improving muscle strength during cancer treatment [25]. Therefore, tailored, supervised resistance training regimens are critical for optimizing muscle health in patients with cancer, as generalized vigorous activity alone is insufficient to induce substantial hypertrophic adaptations.

Misreporting and recall bias in dietary assessment represent another key factor that may explain the lack of association observed between protein intake, physical activity, and ALSTI. In the present analysis, individuals with lower reported protein intake, regardless of physical activity intensity, had significantly higher BMI but paradoxically reported lower energy and protein intake. This pattern is consistent with a well-documented phenomenon, as adults with obesity are more likely to underreport their dietary intake, particularly energy and macronutrients, when using self-report methods such as 24-h dietary recalls [17]. This misreporting can attenuate or obscure true associations between dietary intake and health outcomes, including ALSTI. Recent reviews highlight that commonly used methods, such as 24-h dietary recalls and food records, are subject to both random and systematic error, with underreporting of energy intake averaging 15–20% and being more pronounced in individuals with obesity and those with cancer cachexia [1, 32]. Multiple studies have demonstrated that food frequency questionnaires developed or adapted for populations with cancer can achieve moderate to good validity and reproducibility for estimating energy, macronutrient, and micronutrient intakes when compared to reference methods such as dietary records, 24-h recalls, or biomarkers [23, 24, 28]. Furthermore, differentiating between "normal" and "unusual" days is critical. For example, Brunvoll et al*.* (2022) showed that 26% of days post-surgery in patients with breast cancer were classified as “unusual” and were characterized by significantly lower reported food intake [4].

Across the four groups, reported weekly minutes of moderate and vigorous physical activity were unexpectedly high for a middle-aged population with a cancer history. This finding warrants careful interpretation, as the physical activity data were derived from self-reported questionnaires, which are subject to a high risk of overreporting of both duration and intensity [37]. Multiple studies have demonstrated that self-reported physical activity in cancer survivors and patients consistently overestimates actual activity levels when compared to objective measures such as accelerometery [11, 44]. This overestimation occurs consistently across questionnaires and cancer types, and has been documented in multiple cohorts. This measurement bias has important implications for interpreting associations with clinical outcomes. Overreporting of physical activity can lead to exposure misclassification, attenuate observed associations between physical activity and clinical outcomes, and potentially mask true relationships. Quantitative bias analyses show a low attenuation factor for self-reported physical activity, indicating that its protective associations with cancer outcomes may be substantially underestimated [12, 26]. Furthermore, self-reported measures may be more reflective of light-intensity activity rather than the intended moderate-to-vigorous intensity, further complicating interpretation [38]. Nevertheless, longitudinal studies are warranted to confirm more robust results on the topic, while future interventional studies will help inform future work in this field, particularly by attempting to control sufficiently for physical activity (possibly assessed using objective measures such as accelerometery) and dietary intake [31].

### Evidence from interventional studies

Our findings were based on a mean protein intake of ~ 1.25 vs. ~ 0.60 g/kg/bodyweight, which resonates with clinical trials investigating multimodal prehabilitation in oncology. The PREHAB randomized controlled trial showed that a structured program combining nutrition, resistance training, and psychological support led to reduced postoperative complications and improved functional recovery in patients with colorectal cancer following surgery [27]. In a secondary analysis, higher protein intake (1.5 g/kg/day) was directly correlated with improvements in lean soft tissue and muscle strength (leg press 1 repetition-maximum) [41]. The PRIMe trial, however, highlighted the challenges of achieving very high protein targets (up to 2.0 g/kg/day) during chemotherapy: only a subset of patients met these goals, but those who did maintained muscle mass and physical performance [13]. These observations underscore both the potential and the feasibility challenges of implementing higher protein prescriptions in routine care. Protein supplementation, especially when integrated into multimodal interventions, may represent a practical strategy to attenuate muscle loss [35]. Beyond nutrition alone, oncology care pathways should formally integrate dietitians and physiotherapists early in the disease trajectory to optimize both dietary intake and structured exercise participation [22]. Importantly, many patients, particularly those with fatigue or pre-cachexia, may not tolerate vigorous exercise. Progressive, lower-intensity resistance programs may therefore be more feasible and scalable across a broader spectrum of patients [18]. Collectively, these interventional data contrast with our findings, reinforcing the role of methodological limitations (misreporting, overreporting, absence of resistance-specific activity data) in shaping the present results. This is in line with a scoping review by Orsso et al*.* (2022) [31] analyzing 113 ongoing trials, which highlighted the heterogeneity of nutritional and exercise interventions in oncology, underlining the need for harmonized protocols and standardized body composition measures [33].

### Limitations

Several limitations should be acknowledged when interpreting our findings. First, the cross-sectional design precludes any inference of causality, limiting our ability to determine the directionality or temporal sequence of the observed associations. Second, both physical activity and dietary intake were assessed using self-reported data, which are subject to recall bias and potential misreporting, particularly in individuals with overweight or obesity, possibly attenuating true associations. Third, the role of sex-specific differences in muscle mass and metabolism was not explored in depth, despite evidence suggesting that sarcopenia may manifest differently across sexes in the context of cancer [39]. Fourth, we were unable to account for markers of systemic inflammation (e.g., interleukin-6, tumour necrosis factor-α), which are known contributors to muscle wasting in oncologic populations, especially in more advanced disease stages [40]. The absence of these data may have limited our ability to control for important biological confounders. Fifth, misclassification of muscle mass may also represent a source of bias, as different scaling methods for ALST (e.g., ALST/height^2^ vs. ALST/BMI) may yield divergent associations with outcomes [21]. Sixth, no data on muscle-strengthening activities were available in NHANES, limiting our capacity to isolate the effects of resistance exercise. Seventh, cancer diagnoses in NHANES are self-reported and not clinically verified; as a result, some cases may represent non-melanoma skin cancers, which are typically non-invasive and may differ from other malignancies in their metabolic and clinical impact. Eighth, the restriction to adults aged 40–59 years, adopted to reduce age-related heterogeneity, limits the generalizability of our findings to older adults, in whom muscle outcomes may be influenced by different patterns of sarcopenia, multimorbidity, and anabolic resistance. Finally, the absence of longitudinal follow-up precluded evaluation of ALSTI trajectories and their responsiveness to lifestyle factors. Future research should integrate objective measures of diet and physical activity, sex-stratified analyses, inflammatory biomarkers, and longitudinal designs to provide more definitive insights into the interplay between protein intake, physical activity, and muscle health in cancer populations.

## Conclusions

In this nationally representative sample of US adults aged 40–59 years with a history of cancer, meeting vigorous or moderate physical activity guidelines in conjunction with higher protein intake was not consistently associated with ALSTI. Longitudinal studies and interventional trials are warranted to confirm these findings, elucidate underlying mechanisms, and inform strategies aimed at preserving muscle mass, particularly in older patients with cancer, who are increasingly vulnerable to secondary sarcopenia.

## Supplementary Information

Below is the link to the electronic supplementary material.

Supplementary Material 1 (DOC 83.0 KB)

## Data Availability

Data is available upon request.
